# Immunolipidomics Reveals a Globoside Network During the Resolution of Pro-Inflammatory Response in Human Macrophages

**DOI:** 10.3389/fimmu.2022.926220

**Published:** 2022-06-30

**Authors:** Sneha Muralidharan, Federico Torta, Michelle K. Lin, Antoni Olona, Marta Bagnati, Aida Moreno-Moral, Jeong-Hun Ko, Shanshan Ji, Bo Burla, Markus R. Wenk, Hosana G. Rodrigues, Enrico Petretto, Jacques Behmoaras

**Affiliations:** ^1^ Singapore Lipidomics Incubator, Life Sciences Institute, National University of Singapore, Singapore, Singapore; ^2^ Institute for Stem Cell Science and Regenerative Medicine (inStem), Bangalore, India; ^3^ Precision Medicine Translational Research Programme and Department of Biochemistry, Yong Loo Lin School of Medicine, National University of Singapore, Singapore, Singapore; ^4^ Program in Cardiovascular and Metabolic Disorders (CVMD) and Center for Computational Biology (CCB), Duke NUS Graduate Medical School, Singapore, Singapore; ^5^ Department of Immunology and Inflammation, Centre for Inflammatory Disease, Imperial College London, London, United Kingdom; ^6^ Laboratory of Nutrients and Tissue Repair, School of Applied Sciences, University of Campinas, Limeira, Brazil; ^7^ MRC London Institute of Medical Sciences (LMC), Imperial College, London, United Kingdom; ^8^ Institute for Big Data and Artificial Intelligence in Medicine, School of Science, China Pharmaceutical University, Nanjing, China

**Keywords:** lipidomics, human macrophages, transcriptomics, globosides, network analysis

## Abstract

Toll-like receptor 4 (TLR4)-mediated changes in macrophages reshape intracellular lipid pools to coordinate an effective innate immune response. Although this has been previously well-studied in different model systems, it remains incompletely understood in primary human macrophages. Here we report time-dependent lipidomic and transcriptomic responses to lipopolysaccharide (LPS) in primary human macrophages from healthy donors. We grouped the variation of ~200 individual lipid species measured by LC-MS/MS into eight temporal clusters. Among all other lipids, glycosphingolipids (glycoSP) and cholesteryl esters (CE) showed a sharp increase during the resolution phase (between 8h or 16h post LPS). GlycoSP, belonging to the globoside family (Gb3 and Gb4), showed the greatest inter-individual variability among all lipids quantified. Integrative network analysis between GlycoSP/CE levels and genome-wide transcripts, identified Gb4 d18:1/16:0 and CE 20:4 association with subnetworks enriched for T cell receptor signaling (*PDCD1*, *CD86*, *PTPRC*, *CD247*, *IFNG*) and DC-SIGN signaling (*RAF1*, *CD209*), respectively. Our findings reveal Gb3 and Gb4 globosides as sphingolipids associated with the resolution phase of inflammatory response in human macrophages.

## Introduction

Macrophages are mononuclear phagocytes which respond to external stimuli through coordinated metabolic and immune responses. Bacterial lipopolysaccharides (LPS) are outer surface membrane components present in all gram-negative bacteria that can stimulate strong innate immune responses. LPS can interact with the membrane receptors CD14 and TLR4 and activate the macrophages through a reprogramming of epigenetic, transcriptomic and metabolic states that form the basis of the pro-inflammatory (classical, M1) response (1–3). When incubated with LPS, macrophages show time-dependent changes in gene expression profiles (3) accompanied by a metabolic reprogramming characterized by up-regulation of glycolysis and its related metabolites (2). Exposure to LPS induces an early inflammatory activation (3h-8h) followed by a resolution (8h-24h) phase. The early increase in tricarboxylic acid (TCA) cycle activity in human and mouse macrophages (4–6) is later antagonized by nitric oxide-mediated inactivation of both the TCA cycle and the electron transport chain components in murine macrophages (7). These LPS-induced changes in glycolysis and TCA cycle activity coincide with lipid uptake and intracellular lipid droplet formation that are necessary for the storage of triglycerides and cholesteryl esters (CE) (8–11). Lipid droplets that form in response to LPS are innate immune hubs at the crosstalk of cell metabolism and host defense (12, 13). Thus, lipid metabolism is linked, directly or through other central carbon pathways, to the macrophage immune response and its inhibition can modulate the pro-inflammatory (8, 14, 15) or pro-resolution (16) states of these cells.

The activity of the TCA cycle is closely related to lipid biosynthesis through acetyl-CoA, a rate-limiting metabolite that serves as precursor for all the main lipid classes, including fatty acids, CE, eicosanoids and complex lipids such as glycerolipids, glycerophospholipids and sphingolipids. Fatty acid synthesis is also essential for monocyte-to-macrophage differentiation (17). In response to LPS as a TLR4 agonist, fatty acids synthesis and triglycerides (TG) usage are essential pathways in mediating myeloid cell effector functions such as antigen presentation, host defense and phagocytosis (16, 18–20).

The lipid metabolism-related responses in activated macrophages are complex and time-dependent. The LIPID MAPS consortium and other groups previously studied the dynamics of lipid biosynthesis in murine macrophage cell lines in response to Kdo2–Lipid A (KLA), an active component of LPS (21–25). When exposed to KLA, the RAW264.7 macrophages rapidly increase their eicosanoid production and exhibit a coordinated late response that involves the synthesis of SP and sterol esters (21).

As the current knowledge on the function of lipids during TLR4-dependent macrophage activation is predominantly based on studies involving murine systems (21–23, 25, 26), here we investigate a vast array of lipids and genome-wide expression changes following a time-course activation in human primary macrophages. We combined LC-MS-based lipidomics and RNA-sequencing at 4 different time points following LPS stimulation of macrophages isolated from healthy individuals. We quantified 260 lipids belonging to 18 distinct sub-classes and covered transcriptomics and lipidomics (i.e. immunolipidomics) dynamics that correspond to very early (30 min), pro-inflammatory (3h-8h) and pro-resolution (8h-16h) phases of LPS response in human macrophages.

Our results confirm that LPS promotes *de novo* SP synthesis, with sphingoid bases (SPB) showing the earliest changes, followed by increase in more complex lipid species, such as ceramides and glycosphingolipids (glycoSP). Various lipid species were distributed across different temporal clusters, with the exception of globosides and CE, which were specific to the resolution phase (8h-16h post LPS stimulation). Interestingly, among all lipids, globosides showed the highest inter-individual variability. We then performed a comparative analysis of globosides and CE by integrating their resolution phase production pattern with genome-wide transcriptomics.

## Results

### Temporal Lipidomics in Classically Activated Human Macrophages

Lipid metabolism is central to the response of macrophages to TLR activation (20, 21). Here we describe the temporal variation of the human lipidome in primary monocyte-derived macrophages isolated from 11 healthy donors following activation with LPS (at 30 min, 3h, 8h and 16h). Cellular lipids were extracted at each different time point and 260 molecular species, covering 18 different sub-classes, were measured by mass spectrometry (LC-MS/MS).

**Figure 1:**
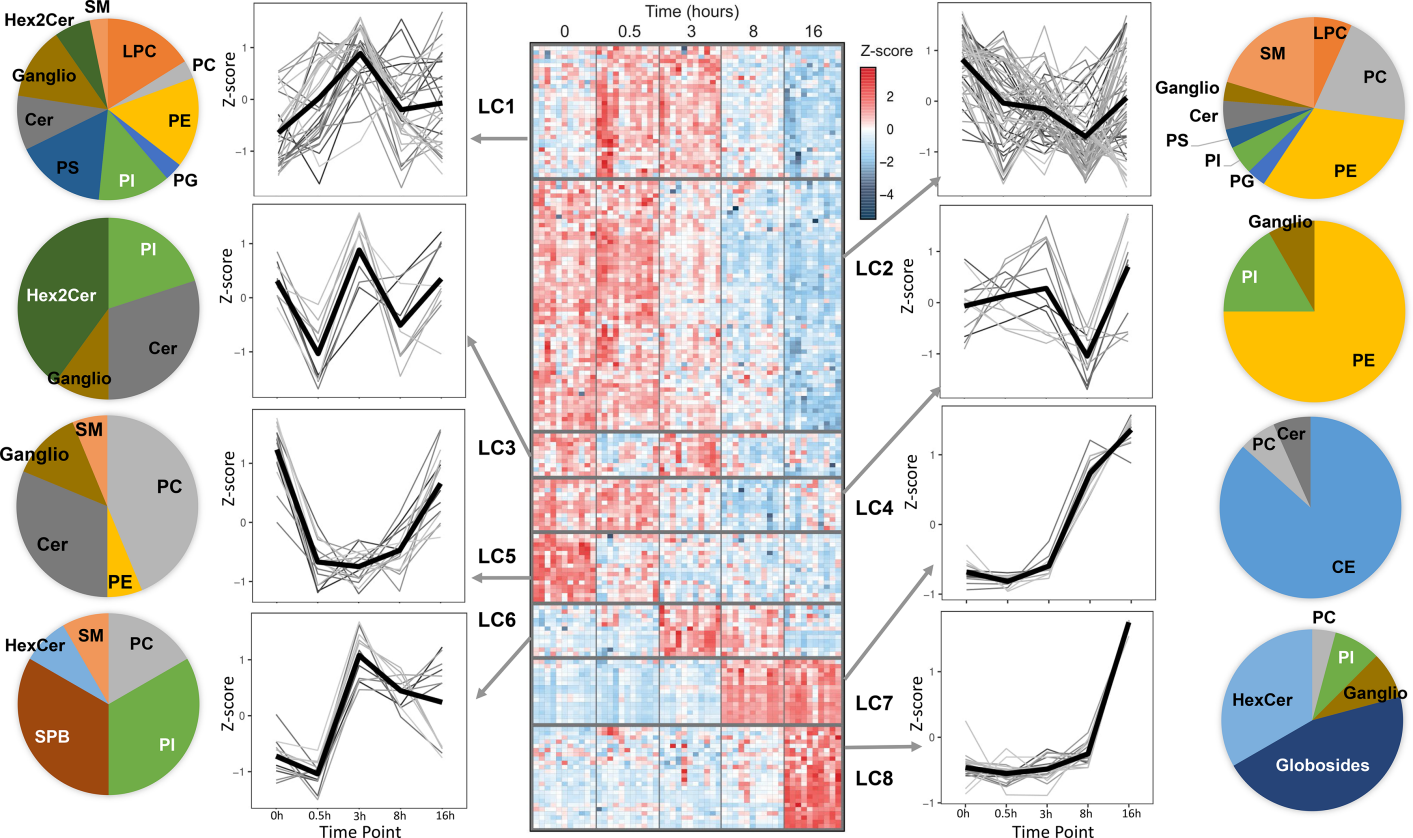
Temporal lipidomics analysis in human macrophages reveal LPS-dependent lipid kinetics. Heatmap showing 179 lipids with significant changes across time (FDR <0.05) are distributed among 8 lipid clusters (LC). Each cluster is separated by horizontal lines. At least N=10 donors were used and hMDMs were used as untreated or LPS-stimulated for 30 min, 3h, 8h and 16h (see *Methods* for cluster generation). In the heatmap, each row depicts the relative abundance of a single lipid and data are presented following z-score transformation. See also **Supplementary Table 1** for details of the lipids included in each time series cluster. The kinetic trends of each LC and the lipid species are represented as line plots and pie charts, respectively. PC, phosphatidylcholines; PE, phosphatidylethanolamines; PI, phosphatidylinositol; PG, phosphatidylglycerols; PS, phosphatidylserine; LPC, lysophosphatidylcholine; Cer, ceramides; SM, sphingomyelins; HexCer, monohexosylceramides; Hex2Cer, dihexosylceramides; CE, cholesteryl esters; Ganglio, gangliosides; SPB, sphingoid bases.

A global overview of the changes in the lipid levels induced by LPS is illustrated in **Figure 1**. The averaged kinetic variations of the lipid molecular species belonging to glycerophospholipids, sphingolipids and cholesteryl esters (CE) were clustered (Lipid Clusters, LC1 to 8) and represented by both heat maps and line charts (**Figure 1**). The lipid composition of the temporal clusters was reported as pie charts. The results show that lipids belonging to the same class can be spread across multiple temporal clusters. These include phosphatidylcholines (PC), phosphatidylethanolamines (PE), phosphatidylinositol (PI), phosphatidylglycerols (PG), phosphatidylserine (PS), lysophosphatidylcholine (LPC), ceramides (Cer), sphingomyelins (SM), monohexosylceramides (HexCer), dihexosylceramides (Hex2Cer) and gangliosides (ganglio). Notably, few sub-classes are exclusively present in single clusters: while CE and globosides belong exclusively to the resolution phase corresponding to LC7 and LC8, sphingoid bases (SPB) are part of LC6, which corresponds to a pro-inflammatory phase cluster that includes lipids and pro-inflammatory genes (*TNF*, *IL12B, CCL3*) peaking at 3h following LPS stimulation (**Figures 1**, **S1**). In addition to SPB, LC6 also contains polyunsaturated fatty acid (PUFA)-containing glycerophospholipids (**Supplementary Table 1**). Furthermore, down-regulation of specific lipid clusters as early as 30 min (LC3 and LC5) suggests post-transcriptional regulation of their production. The early and sharp down-regulation is more prominent in LC5 that contains a relatively large proportion of PC and Cer (**Figure 1**). As part of secreted bioactive lipids, we measured the media levels of 5 prostaglandins (PGE2, PGF2, PGJ2, PGD2, 15d-PGD2), 3 thromboxanes (TXB1, TXB2, TXB3), 1 leukotriene (20-OH-LTB), as well as arachidonic acid (AA), docosahexaenoic acid (DHA) and eicosapentaenoic acid (EPA, **Figure S2**). As expected, most of the eicosanoids are produced as a response to LPS (**Figure S2**). AA, DHA and EPA were detectable in basal human macrophages and only EPA levels were up-regulated significantly upon LPS treatment (**Figure S2**).

Among other clusters, LC7 and LC8 include CE and globosides, which (i) are specific to these clusters and (ii) increase sharply between 8h-16h following LPS stimulation. This suggests their involvement in the resolution phase of pro-inflammatory response to TLR4 activation.

### Inter-Individual Variation in the Lipidomic Response in Human Macrophages

**Figure 2:**
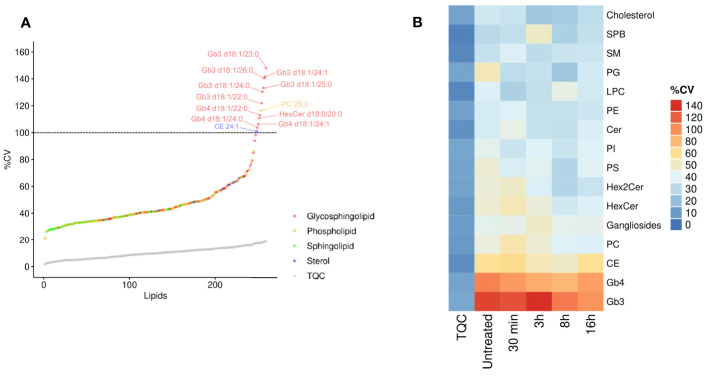
Resolution phase lipids show high inter-individual variability. **(A)** The coefficient of variation (CV%) was calculated for each lipid for untreated hMDMs. The CV% of technical quality controls (TQC) is shown as grey circles. Dashed line represents 100% CV; Macrophages derived from N=11 donors. **(B)** The CV% values were calculated for each lipid class and are color coded. The first column (TQC) indicates the technical variation for each lipid class. PC, phosphatidylcholines; PE, phosphatidylethanolamines; PI, phosphatidylinositol; PG, phosphatidylglycerols; PS, phosphatidylserine; LPC, lysophosphatidylcholine; Cer, ceramides; SM, sphingomyelins; HexCer, monohexosylceramides; Hex2Cer, dihexosylceramides; CE, cholesteryl esters; SPB, sphingoid bases; Gb3, globotriaosylceramide; Gb4, globotetraosylceramide.

We hypothesized that inter-individual variation can influence the specificity of the lipidomic responses in human macrophages. We thus interrogated whether the variability in the intracellular levels of lipid species and classes are randomly observed throughout all the quantified lipids. Using the LC-MS/MS technical quality control (TQC) as an estimation of technical variability, we measured the biological variability in lipid species in primary macrophages from 11 healthy donors. Strikingly, when plotted across 260 lipids quantified in human macrophages, the coefficient of variation (CV) of globotriaosylceramide (Gb3) and globotetraosylceramide (Gb4) showed the highest %CV (**Figures 2A, B** and **Supplementary Table 2**), suggesting that, among others, the LPS-induced production of this lipid family might be under genetic control. Specifically, all the 6 Gb3 lipid species were among the lipids that showed the highest %CV (**Figure 2A**). Gb3 and Gb4 belong to globosides, which are glycosphingolipids (glycoSP) that are up-regulated during the resolution phase of the pro-inflammatory response (**Figure 1**). Accordingly, CE, which show a similar kinetic pattern to the one observed with globosides (**Figure 1**), are among the lipid species that show the second highest variability between individuals (**Figure 2B**). Taken together, these results show that lipid species such as globosides and CE that uniquely belong to the resolution phase (8h-16h following LPS; **Figure 1**), also show relatively high biological variability in their biosynthesis in basal (untreated) and LPS-stimulated states (**Figure 2B**). These results suggest functional and heritable relevance of globosides in the regulation of TLR4 pathway.

### Temporally Regulated Sphingolipid Production in LPS-Stimulated Human Macrophages

We measured 12 species of globosides: 8 Gb3 and 4 Gb4 species (**Supplementary Table 1**) that, to our knowledge, have not been previously quantified at this level of molecular and temporal resolution in primary human macrophages. Given the synthesis of these glycoSP species during the resolution phase, we next investigated the kinetics of their precursors (i.e. ceramides), as well as other sphingolipid species. We quantified 28 species of ceramides, with N-acyl chain lengths ranging from 14 to 26 carbons and a total number of double bonds from 0 to 2. We also report the specific variation of low abundant ceramide species containing d16:1 and d18:2 (diene) SPB. The specific biological function of these dienes-containing sphingolipids is still unknown and the enzymatic step that generates these molecules was only recently described (27). In order to establish a comprehensive description of the sphingolipidome, we also measured 4 SPB, 17 HexCer, 6 Hex2Cer, 21 gangliosides and 29 SM.

**Figure 3:**
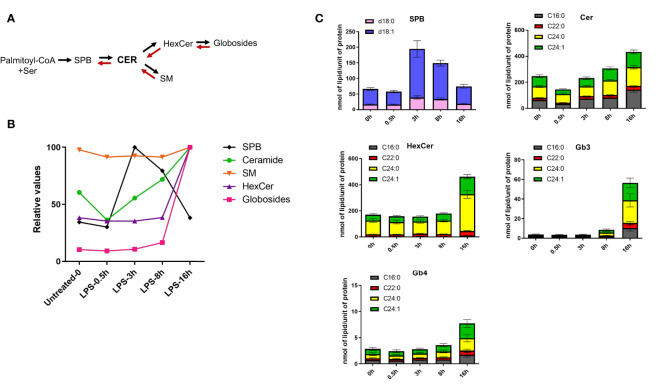
LPS stimulation induces *de novo* sphingolipid synthesis in human macrophages. **(A)** The simplified schematics of sphingolipid biosynthesis. **(B)** LPS-induced kinetics for sphingoid bases (SPB), ceramides, sphingomyelins (SM), monohexosylceramides (HexCer) and globosides (Gb3 and Gb4). Relative values were calculated using the mean of each lipid class across the time-points. **(C)** Quantification of different sphingolipid species throughout the LPS-time course in human macrophages. SBP represent sphingosine (d18:1, blue) and sphinganine (d18:0, pink). All the ceramides, HexCer and globosides (Gb3 and Gb4) shown in the figure contain a d18:1 sphingoid base, while the fatty acyl chains (C16:0, C22:0, C24:0, C24:1) are indicated in different colors. Error bars are s.e.m.

The first step of the *de novo* sphingolipid synthesis is catalyzed by serine palmitoyltransferase (SPT), an enzymatic complex consisting of multiple subunits that generates SPB d18:0 from serine and palmitate (**Figures S3**, **3A**). LPS induces time-dependent changes in the expression levels of almost all enzymes implicated in the sphingolipid and glycoSP metabolic pathways (**Figure S3**). When clustering the kinetics of lipid changes, all four SPB (d16:1, d18:0, d18:1, d18:2) were found in LC6, as their respective concentration peaked between 3-8h and returned to the basal levels at 16h (**Figures 1**, **3B, C** and **Supplementary Table 1**). The Cer, either containing d18:1 or d18:2 backbones are spread across clusters LC1, 2, 3, 5 and 7 (**Figure 1** and **Supplementary Table 1**). Among Cer, most of the species decrease in abundance shortly after LPS treatment (30 min) and increase progressively at later stages (**Figures 3B, C**). Most of the SM showed no major changes in their concentration during the LPS time course, although their minimal decrease at 3h might contribute to the corresponding increase in ceramide levels (**Figure 3B**). The more complex sphingolipids, such as HexCer, only increased at 16h (**Figures 3B, C**). Strikingly, all the globosides clustered together in LC8 (**Figure 1**) and showed a large accumulation at 16h (**Figures 3B, C**). Taken together, these results suggest that LPS induces *de novo* synthesis of sphingolipids, which lead, among others, to the production of globosides that control the resolution phase (8h-16h) of pro-inflammatory response. LPS-induced *de novo* synthesis of glycoSP is consistent with a previous report showing a role of SREBP1 during the resolution phase dynamics (16). *SREBF1* (encoding SREBP1) show a biphasic mRNA activation upon stimulation with LPS in human macrophages (**Figure S1**). In parallel with the *de novo* synthesis of sphingolipids, we observed an increase in the concentration of unsaturated SPB species coinciding with a temporary decrease of their ceramide precursors (**Figure 3C**). This suggests a partial involvement of the salvage pathway, rapidly degrading SM and ceramides to generate SPB species (28).

### The Resolution Phase Lipidome and Its Association With Human Macrophage Transcriptome

**Figure 4:**
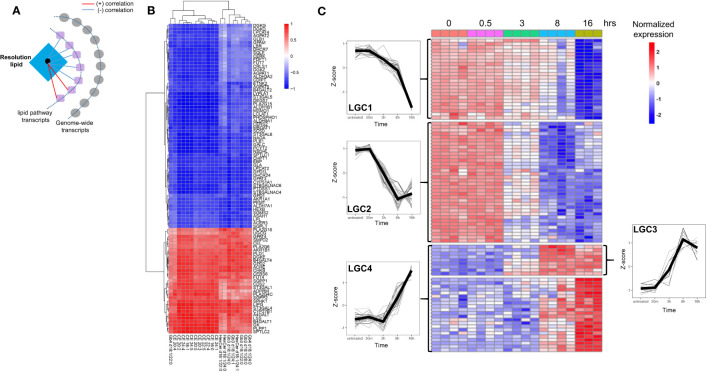
The resolution phase lipids show strong correlation with lipid pathway genes. **(A)** Schematic for the correlation analysis. **(B)** Heatmap showing the correlation of the resolution lipids and lipid-pathway related genes. **(C)** Heatmap showing the kinetics of lipid-pathway-related genes throughout the LPS time-course. The lipid gene clusters (LGC) are separated by horizontal lines. At least n=3 samples for untreated (0), 30 min (0.5h), 3h, 8h and 16h LPS treatment.

Because of their relatively high biological variability and unique temporal pattern, we next focused on the resolution phase-associated lipids and their potential regulation by transcriptional networks. Prior to integrating the resolution phase lipids with genome-wide transcriptomics, we focused on lipid pathway-related transcripts and their potential correlation (**Figure 4A**). This was aimed to create a core lipid vs. lipid-gene association that can be used for genome-wide network-based approaches based on partial correlation. Pearson correlation between the 39 resolution lipid species belonging to LC7 and LC8, and 182 lipid pathway-related transcripts was computed (see *Methods*). After filtering, 91 transcripts were robustly associated with 20 of the resolution phase lipids (**Figures 4B, C**; see *Methods*). Among these, some transcripts showed positive and others negative correlation with 20 resolution phase lipid species (**Figure 4B**). Of note, *A4GALT*, the gene responsible for the synthesis of globosides, was among the most strongly positively correlated genes. Other transcripts such as those that regulate cholesterol biosynthesis (*SQLE*, the rate-limiting enzyme), showed a negative correlation (**Figure 4B**). The time-dependent clustering of the expression levels of these 91 transcripts shows that members of lipid gene cluster 3 (LGC3) and LGC4 increased their expression at 8 and 16 h, with a kinetic pattern similar to the resolution phase lipids previously clustered in LC7 and LC8 (**Figure 1**). Conversely, LGC1 and LGC2 showed a reduced expression at the same late time points (**Figure 4C**). In summary, this analysis showed that resolution phase lipids strongly correlate with lipid pathway transcripts, allowing us to identify a core ‘lipid/transcript’ association that we next used for integration involving genome-wide transcriptomics in LPS-stimulated hMDMs.

**Figure 5:**
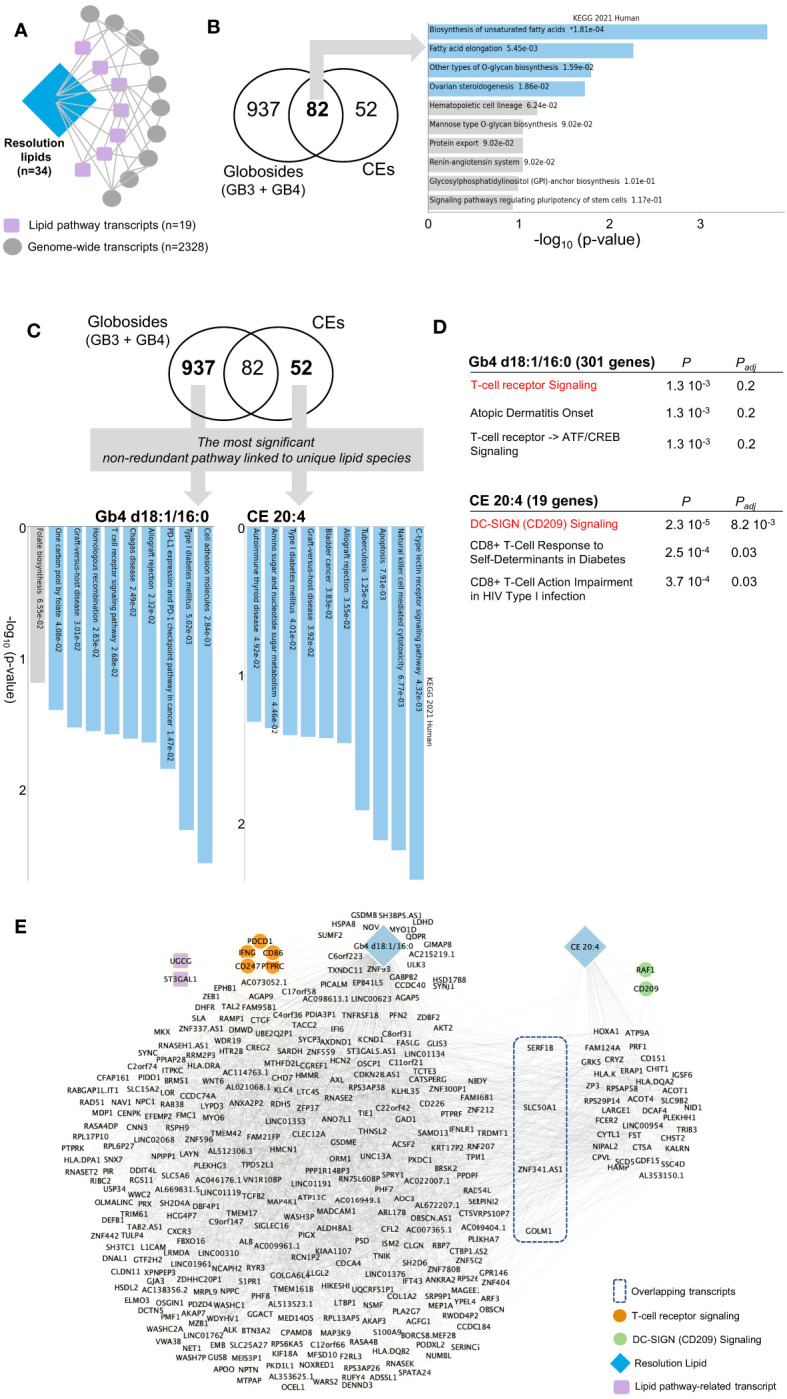
Network analysis by partial correlation identifies specific pathways associated with the resolution phase lipids. **(A)** Schematic for partial correlation between resolution lipids, lipid pathway transcripts and genome-wide transcripts. The n denotes the number of genes/lipids retained after the filtering process applied in partial correlation. **(B)** Venn diagram showing partially correlated genes with globosides (Gb3 and Gb4) and cholesteryl esters (CE). Pathway analysis on the shared 82 genes is shown on the right panel. **(C)** Venn diagram highlighting the most significant non-redundant pathway in globosides and CE. KEGG pathways are shown on the panel below. **(D)** Elsevier Pathways for Gb4 d18:1/16:0 and CE 20:4. P and P_adj_ denote the P-value and the adjusted P-value, respectively. **(E)** Network representation based on partial correlation for Gb4 d18:1/16:0 and CE 20:4. For panels **(B, C)**, the bar chart shows the top 10 enriched terms in the chosen library, along with their corresponding p-values. Colored bars correspond to terms with significant p-values (<0.05). * next to a p-value indicates the term also has a significant adjusted p-value (<0.05).

We thus extended the immunolipidomic analysis of the resolution phase to the entire transcriptomic dataset (**Figure 5A**). First, Pearson correlation between these 91 lipid-pathway related gene transcripts (**Figure 4B**) and genome-wide transcripts was computed. Following partial correlation analysis (to identify relevant associations), 34 lipids, 19 lipid pathway-related transcripts and 2328 genome-wide transcripts were retained (**Figure 5A**, see *Methods*). Among the resolution lipids, globosides and CE showed the highest inter-individual variability (**Figures 2A, B**), suggesting their potential regulatory effects on LPS/TLR4 in macrophages (29). We thus focused on these lipid species and hypothesized that specific gene networks could be associated with their production in human macrophages. Comparative analysis of both globoside subspecies (Gb3 and Gb4) and CE showed 937 and 52 transcripts respectively, that specifically correlate with each lipid class (**Figure 5B**; **Supplementary Table 3**). Interestingly, the 82 commonly associated transcripts are enriched for ‘biosynthesis of unsaturated fatty acids’, confirming that lipid biosynthesis pathway is conserved between globosides and CE (**Figure 5B**). In order to identify unique pathways linked to each lipid, we looked for the most significant non-redundant pathways. Despite the different levels of statistical significance, we found specific pathways that correlated with two lipid species: Gb4 d18:1/16:0 was associated with cell adhesion molecules, while CE 20:4 was enriched for C type lectin receptor signaling pathway (**Figure 5C**). More specifically, Gb4 d18:1/16:0 and CE 20:4 were associated with ‘T cell receptor signaling’ and ‘DC-SIGN (CD209) signaling’, respectively (**Figure 5D**). The respective networks associated with Gb4 d18:1/16:0 and CE 20:4 show the genes belonging to T cell receptor (*PDCD1*, *CD86*, *PTPRC*, *CD247*, *IFNG*) and DC-SIGN (*RAF1*, *CD209*) signaling pathways, respectively (**Figure 5E**). Interestingly, the Gb4 d18:1/16:0 network also contain *UGCG*, which catalyzes the first glycosylation step in the biosynthesis of glycoSP. This suggests the specificity of the gene network associated with globoside production in macrophages.

## Discussion

In macrophages, LPS/TLR4-mediated reprogramming of lipid homeostasis can have two major consequences. Evidently, the macrophage uses lipid biosynthesis to mount an effective pro-inflammatory response and previous studies show how this crosstalk between lipidomic and immune responses (i.e. immunolipidomics) occur at the level of individual lipids and/or as part of lipid droplets (8, 12, 13, 16, 20). A less studied but equally important aspect, is the usage of specific lipids such as sphingolipids by the macrophage to regulate the ongoing TLR4 trafficking and signalling events and the evolution of intracellular pathways that shift from pro-inflammatory (3h-8h) to resolution (8h-24h) phases (29, 30). Hence reprogramming of lipid metabolism coordinate regulatory and effector pathways in LPS-stimulated macrophages.

Systems-level analysis of lipids and their dynamic behavior has been pivotal in understanding human disease (31, 32) and mapping lipidome in human cells has revealed the heterogeneity of lipidome arising from inter-individual variability (33). Here we describe the immunolipidomics profiling of human monocyte-derived macrophages following their activation with LPS. By using LC-MS/MS and RNA-seq analyses, we describe LPS-mediated patterns of activation in specific classes of lipids and transcripts. Our results show how pro-inflammatory and resolution phases of activation depend on reprogramming of lipid metabolism in human macrophages. We also observed that some lipid sub-classes were not affected by the LPS stimulation and these include most of the molecular species of PC and PE in agreement with what was shown previously in murine models (21).

Our results represent a comprehensive lipidomic profiling of human macrophages. Indeed, while most lipid classes follow indistinctive kinetics, sphingolipid classes show clear patterns during pro-inflammatory and resolution phases following the LPS response. This could be explained by the LPS-induced *de novo* biosynthesis of fatty acids, which produce palmitate as a building block of sphingolipids in macrophages (29). It is therefore not surprising that the LPS-induced *de novo* synthesis of sphingolipids follows well-defined kinetics in human macrophages. For instance, all sphingoid base species (SPB, the precursor of ceramides), peak during the pro-inflammatory phase (3h), while their products (ceramides and their derivatives - HexCer and globosides) accumulate during the resolution phase. The early production of SPB upon TLR4 activation is in line with previous reports (25) and the late production of ceramide-derived sphingolipids is due to their relative structural complexity that requires additional enzymatic steps (29). Our results also show temporal differences in the production of sphingolipid subclasses, suggesting that the fatty acid chain length of each lipid specie affects their kinetics following LPS treatment. For instance, HexCer containing very long chain fatty acyls (C22-26) were up-regulated during the resolution phase, while only one HexCer (C18:0) belonged to the pro-inflammatory phase, which may indicate a specific role of this sphingolipid during the acute phase of LPS response.

The phase-dependent production of sphingolipids may be linked to the transcriptional control of regulatory genes. LXRs and SREBPs (mainly SREBP1 encoded by *SREBF1*) cooperate during the positive regulation of fatty acid biosynthesis (34–36). During the resolution phase of TLR4-mediated macrophage activation, SREBP1 activates the genes involved in the biosynthesis of unsaturated fatty acids (16), including palmitate used in the *de novo* sphingolipid biosynthesis. Thus, SREBP1 activation may be linked to the production of complex sphingolipids during the resolution phase of TLR4 activation and the LPS-mediated biphasic regulation of *SREBF1* expression is in agreement with this. Furthermore, it was proposed that sphingolipids themselves can modulate SREBP-dependent broader lipid biosynthesis (37) and membrane lipid composition (30).

Our results pointed toward resolution phase lipids being predominantly composed of CE and globosides. CE accumulate during the resolution phase (8h-16h) while free cholesterol measurements did not show differences between time points after LPS treatment. This suggests that a continuous synthesis of CE is required to avoid any accumulation of unesterified cholesterol in macrophages. CE are part of lipid droplets and their LPS-induced production and storage in mononuclear phagocytes is well known (2, 8–11, 19, 21, 38, 39). Blocking cholesterol synthesis results in unresolved wound healing in the skin with increased macrophage infiltration (40). Our integrative network analysis pointed toward a potential association of specific cholesteryl ester species (CE 20:4) with DC-SIGN signalling during the resolution phase of LPS response. Interestingly, DC-SIGN and unesterified membrane cholesterol are pivotal for macrophage-mediated infection of CD4+ T cells (41).

Here, we provide a comprehensive measurement of globosides and show a significant accumulation of these neutral glycoSP during the resolution phase of LPS-response in healthy individuals. Strikingly, 9 out of 12 lipid species that showed the highest biological variability, are globosides with the entire Gb3 family being among the ones that showed the highest % CV. While we report Gb3 and Gb4 species in human macrophages, Gb5 (also known stage-specific embryonic antigen-3 or SSEA3) have not been quantified as the LC-MS/MS-based detection of this globoside may be misapprehended due to existence of isobaric lipids. However, the up-regulation of Gb5 precursors (Gb3 and Gb4) during the resolution phase may suggest a possible role of Gb5 in human macrophages. Similarly, Gb4 and (n)Lc4 which belong to lacto/neolacto-type glycosphingolipids show identical m/z values, which may indicate the involvement of neolacto-series pathway. However, the LPS-induced robust increase of *A4GALT* expression and Gb3 (i.e. the only precursor of Gb4) during the resolution phase argues in favor of Gb4 quantification in human macrophages. The inter-individual variability in Gb3 and Gb4 globosides is conserved in basal and during the time course of LPS stimulation, suggesting that the level of these lipids are controlled by quantitative trait loci during homeostatic conditions in macrophages.

Besides their significant increase during the resolution phase of the LPS response, biological variability of globosides suggests that these glycoSP can contribute to macrophage effector functions, such as antigen presentation while also regulating the TLR4 signalling. The LPS response in macrophages is characterized by decreased cell division (42, 43), progressive changes in mTOR-mediated lysosome morphology and formation of autophagosomes (25, 44, 45). These sequential phenotypic changes lead to a resolution phase characterized by the expression of genes involved in antigen presentation such as CD86 (46). In THP-1 monocytic cells, 24h LPS stimulation causes the up-regulation of proteins associated with antigen presentation and T cell stimulation together with alterations in intracellular vesicular and endosomal trafficking (47). Antigen presentation is an effector function less studied in macrophages compared to dendritic cells (48). A potential role of globoside species in antigen presentation during the resolution phase can be hypothesized based on several lines of evidence. First, glycoSP production accompanies monocyte/macrophage differentiation (49), during which the cells acquire their antigen-presenting properties. Second, our network results show an association between Gb4 d18:1/16:0 and T cell signalling, which may indicate a potential regulatory role of Gb4 lipid species in antigen presentation (*CD86*) and T cell activation (*PDCD1*). Furthermore, other glycoSP that belong to the ganglioside family can activate iNKT cells which are lipid-reactive T cells (50). Finally, globosides themselves are antigens on red blood cells in the P blood group system [known as P1PK (51)]. It is therefore plausible that biological variability in the production of globosides may contribute to the inter-individual variability underlying the heterogeneity of antigen cross-presentation by macrophages. In addition to inter-individual variability, globosides contribute cell-to-cell variation that define fibroblast subpopulations (52). In terms of the potential interaction of globosides with the TLR4 complex at the membrane, current evidence suggests both agonist (53) and inhibitory (54) effects, which require further investigation (29). Nevertheless, it is tempting to hypothesize a stratification of healthy individuals as ‘efficient’ or ‘inefficient’ LPS responders based on the highly variable globoside profiling and its correlation with resolution phase transcripts. Such analyses can be combined with functional assays, in order to draw definitive conclusions whether specific globoside species can be used as markers of the macrophage resolution phase in response to LPS. Although our study is limited by the analysis of a relatively small sample size, macrophage responses to LPS have been previously used as a proxy for susceptibility to human macrophage-related pathology (55).

In summary, our immunolipidomic analysis reveals time-dependent sphingolipidome dynamics which result in the up-regulation of globosides during the resolution phase of TLR4 response. Given their inter-individual variability and possible regulatory role on TLR4 signalling and antigen presentation, we propose that Gb3 and Gb4 globosides may be targeted during the diseases characterized by M2-like (anti-inflammatory, resolution, repair) macrophage activation. Alternatively, their quantification in genetically outbred populations may be used as a proxy for determining the efficacy of the resolution phase phenotypes (i.e. T cell activation) in macrophages. A detailed globoside quantification may add value to existing surrogates for defining macrophage polarization in human inflammatory disease.

## Methods

### Isolation of Human Primary Macrophages

Human MDMs from healthy donors were differentiated from buffy cones using gradient separation (Histopaque 1077, Sigma) and adhesion purification. Following Histopaque separation, peripheral blood mononuclear cells were resuspended in RPMI (Life Technologies), and monocytes were purified by adherence for 1 hour at 37°C, 5% CO2. The monolayer was washed three times with Hank’s Balance Salt Solution (HBSS) to remove non-adherent cells, and monocytes were matured for 5 days in RPMI containing 100 ng/mL macrophage colony-stimulating factor (M-CSF) (PeproTech, London, UK) and 10% fetal calf serum (Labtech International). Human MDMs were treated with LPS (Escherichia coli serotype O111:B4; Cat No L4391; 100 ng/ml) for 30min, 3h, 8h, 16h or left untreated (basal).

### RNA Extraction and Library Preparation

Total RNA was extracted from MDMs using Trizol (Invitrogen) and RNeasy mini kit (Qiagen) according to manufacturer’s instructions, with an additional purification step by on-column DNase treatment using the RNase-free DNase Kit (Qiagen) to ensure elimination of any genomic DNA. The integrity and quantity of total RNA was determined using a NanoDrop 1000 spectrophotometer (Thermo Fisher Scientific) and Agilent 2100 Bioanalyzer (Agilent Technologies). 500 ng of total RNA was used to generate RNA-seq libraries using NEBNext Ultra II Directional RNA Library Prep kit for Illumina, according to the manufacturer’s instructions. Briefly, RNA was purified and fragmented using poly-T oligo-attached magnetic beads using two rounds of purification followed by the first and second cDNA strand synthesis. Next, cDNA 3’ ends were adenylated and adapters ligated followed by 11 cycles of library amplification. Finally, the libraries were size selected using AMPure XP Beads (Beckman Coulter) purified and their quality was checked using Agilent 2100 Bioanalyzer. Samples were randomized to avoid batch effects and multiplexed libraries were run on a single lane (8 samples/lane) of the HiSeq 2500 platform (Illumina) to generate 100bp paired-end reads. Expression levels are displayed as Log2 TPM. The change in expression across time was statistically assessed using a likelihood ratio test and false discovery rate (LRT_FDR). These were computed using the R package DESeq2 (56).

### Lipid Extraction

Lipids were extracted from the cell pellets using single-phase butanol methanol extraction. 200 µL of Butanol : Methanol (1:1) containing internal standards (5 µL SPLASH^®^ LIPIDOMIX^®^ Mass Spec Standard and 5 µL of Cer/Sph Mixture I) were added to the cell pellets. The samples were then vortexed, sonicated for 30 minutes and centrifuged for 10 minutes at 14,000 g at 25°C. The supernatant was transferred into new Eppendorf safe-lock tubes. 60 µL were transferred into MS vials and 20 µL of lipid extract from each sample were pooled to create technical quality control (TQC) samples. The TQC was injected at the beginning, in intervals of 10 samples, and at the end of the analysis.

### Eicosanoid Quantification

The cell culture media was used for the eicosanoid analysis. The media was collected from untreated (basal) and 16h LPS-treated human macrophages. Samples were extracted using Strata™-X 33 µm Polymeric Reversed Phase extraction columns (8B-S100-UBJ, Phenomenex). The columns were conditioned with 3 mL of 100% MeOH and equilibrated with 3 mL of H2O. 1 mL of media was loaded and the columns were washed with H_2_O: MeOH: acetic acid (90:10:0.1, v/v/v) to remove impurities, and the metabolites were then eluted with 2 times 500 µL of 100% MeOH. The eluent was dried and resuspended in 100 µl of water-acetonitrile-acetic acid (60:40:0.02, v/v/v). The samples were then analyzed based on the protocol adapted from Ambaw and colleagues (57).

### Targeted Lipidomics

All the lipid extracts were analyzed using Agilent 1290 UHPLC coupled with Agilent 6495 triple quadrupole mass spectrometer. Sphingolipids were analyzed using previously described LC-MS methods (58, 59). The mobile phase A was prepared by mixing 600 mL methanol, 400 mL water, 2 mL formic acid and 1.33 mL ammonium acetate solution (7.5M). The mobile phase B was prepared by mixing 600 mL methanol, 400 mL isopropanol, 2 mL formic acid and 1.33 mL ammonium acetate solution (7.5 M). The mobile phases were mixed and sonicated for 10 min before use. A 2 μL sample was injected into the Agilent ZORBAX RRHD Eclipse Plus C18 (2.1 x 100mm, 1.8 µm) column, thermostatted at 40°C. Gradient elution was performed at a flow rate of 400 μL/min, starting at 0% B to 10% B over 3.0 min, 40% B at 5.0 min, 55% B at 5.30 min, 60% B at 8.0 min, 80% B at 8.50 min, held at 80% B until 10.5 min, 90% B at 16.0 min, held at 90% B until 19.0 min, 100% B at 22.0 min and re-equilibrated at 0% B from 22.1 min to 25.0 min. The total run time was 25.0 min.

The LC effluent was introduced into Agilent 6495 QQQ mass spectrometer *via* an AJS ESI ion source operating under the following conditions: gas temperature, 200°C; gas flow, 15 L/min; nebulizer, 25 psi; sheath gas heater, 200 arbitrary units; sheath gas flow, 12 arbitrary units; capillary, 3500 V; Vcharging, 500 arbitrary units. Positive high/low pressure RF of the iFunnel were set to 210/110. The mass spectrometer operated in positive polarity mode.

For glycosphingolipids (glycoSP), the mobile phase A was water/acetonitrile (60/40, v/v) and mobile phase B was 2-propanol/acetonitrile (90/10, v/v), with both containing 10 mM of ammonium formate. The mobile phases were mixed and sonicated for 10 min prior to their use. A 2 µL sample was injected onto the Agilent ZORBAX RRHD Eclipse Plus C18, 95 Å, 2.1 x 50mm, 1.8 µm UPLC column, thermostatted at 40°C. Gradient elution was performed at a flow rate of 400 μL/min starting at 0% B to 60% B over 2.0 min, 100% B at 7.0 min, held at 100% B until 9.0 min and re-equilibrated at 20% B from 9.01 min to 10.8 min. The total run time was 10.8 min.

The LC effluent was introduced into Agilent 6495 QQQ mass spectrometer *via* an AJS ESI ion source operating under the following conditions: gas temperature, 200°C; gas flow, 12 L/min; nebulizer, 25 psi; sheath gas heater, 250 arbitrary units; sheath gas flow, 12 arbitrary units; capillary, 3500 V; Vcharging, (+) 500 and (-) 1500 arbitrary units. Positive high/low pressure RF of the iFunnel were set to 210/110 and negative high/low pressure RF of the iFunnel were set to 150/60. The mass spectrometer operated in both positive and negative polarity mode.

Glycerophospholipids and CE were analyzed based on methods described by Alshehry et al. (60). The mobile phases, column, gradient and injection volume were the same as used for glycoSP analysis.

The LC effluent was introduced into Agilent 6495 QQQ mass spectrometer *via* an AJS ESI ion source operating under the following conditions: gas temperature, 200°C; gas flow, 12 L/min; nebulizer, 25 psi; sheath gas heater, 250 arbitrary units; sheath gas flow, 12 arbitrary units; capillary, 3500 V; Vcharging, 500 arbitrary units. Positive high/low pressure RF of the iFunnel were set to 150/60. The mass spectrometer operated in positive polarity mode.

Eicosanoids were analyzed based on methods described by Ambaw and colleagues (57). The mobile phase A was acetonitrile/water/acetic acid (60/40/0.02, v/v/v) and mobile phase B was acetonitrile/isopropanol (50/50, v/v). A 10 µL sample was injected onto the Acquity UPLC BEH shield RP18 column (2.1 × 100 mm; 1.7 m; Waters), thermostatted at 40°C. Gradient elution was performed at a flow rate of 500 μL/min starting at 1% B to 10% B over 1.0 min, 55% B at 5.0 min, 99% B at 5.5 min, held at 99% B until 6.0 min, and re-equilibrated at 1% B from 6.5 min to 10.0 min. The total run time was 10 min.

The LC effluent was introduced into Agilent 6495 QQQ mass spectrometer *via* an AJS ESI ion source operating under the following conditions: gas temperature, 260°C; gas flow, 14 L/min; nebulizer, 35 psi; sheath gas heater, 250 arbitrary units; sheath gas flow, 12 arbitrary units; capillary, (+) 3500 V and (-) 4000 V; Vcharging, (+) 1500 arbitrary units and (-) 1000 arbitrary units. Positive and negative high/low pressure RF of the iFunnel were set to 150/60. The mass spectrometer operated in negative polarity mode.

### Lipid Identification and Quantification

The data were processed with the Agilent MassHunter Quantitative Analysis Software Version 8. Lipids were identified based on retention time and MRM transitions. Concentrations of lipids were obtained by normalizing the lipid peak areas to those of the class-specific internal standard and the total protein levels. Endogenous species were quantified using one standard per lipid class, providing relative quantitation results. For analytical quality assurance, we quantified lipids that could only be reproducibly detected [coefficient of variation (CV) <30%] and that were not present in the blank extracts (<10% of QC).

### Time Series Clustering of Lipids

Abundances of 260 lipid species were measured in biological replicates at several time points (untreated, 30 min, 3h, 8h, 16h). Data were quantile normalized by using the function normalize.quantiles from R package preprocessCore. Lipid abundances were logged (log2 after adding an offset of 1) and differential lipids across time were computed with the R package limma (61). Differential lipids were computed both pairwise (comparisons: 30 min vs untreated, 3h vs 30min, 8h vs 3h and 16h vs 8h) and across all time points. Donor sample ID was included in the differential model to account for inter-individual effects. False discovery rate (FDR) of the differential tests was computed using the Benjamini & Hochberg (BH) method implemented in the limma package function topTableF. Lipids with significant changes across time (FDR < 0.05, 208 lipids) were retained for time series clustering.

GPClust (62, 63) was used to carry out time-course clustering. GPClust clusters time series data by simultaneously fitting Gaussian processes (GP) to observed expression profiles and estimating the number of clusters required to fit the data. Each cluster proposed by GPClust is a GP defined by an estimated mean and covariance function, which can be approximated using a multivariate Gaussian distribution.

Lipid abundances were provided to GPclust after adjusting for inter-individual effects (donor sample ID) by taking the residuals of a linear model in which the quantile-normalized logged lipid abundances were explained by the donor sample ID. Lipid abundances were then z-scored and time was squared root+2 transformed. Thirty GPClust runs were carried out, ten with each alpha value (0.00001, 0.0001, 0.001, 0.001 and 0.1 alpha values were used). Lengthscale parameter was set to 6. From GPClust output, each lipid was assigned to the module with the highest probability of module membership. To select the most representative clustering, the pairwise variance of information distance was computed across all runs by using the function vi.dist from R package mcclust (version 1.0). Within each alpha (10 runs), the mean of the clustering distances was computed and the clustering run with the smallest mean was selected. This resulted in 14 clusters among which 8 clusters had at least 10 lipids. For representation purposes, the modules were further clustered to highlight major patterns in the data (modules with less than 10 lipids were deemed as noise). Pairwise symmetric Kullback-Leibler (KL) divergence was computed between all clusters (using the 0-16 hours mean and covariance matrix of the multivariate Gaussian distributions which approximates the underlying GP). KL divergence was computed using the function kl.norm from R package monomvn 1.9-7. The computed divergences were converted into a distance matrix using the R function as.dist and hierarchical clustered using the R function hclust using the agglomeration method “complete”. The obtained order was retrieved to display the lipidomics time course in a single heatmap where the individual values of lipids are shown in each cluster.

### Lipid-Transcript Correlation and Network Analysis

Pearson correlation between the 39 lipids from clusters LC7 and LC8 in the resolution phase and 182 lipid pathway-related gene transcripts (KPGminer https://doi.org/10.1101/416131) was computed and p-values were adjusted by Benjamini-Hochberg correction. The lipid pathways include sphingolipid metabolism, glycoSP biosynthesis (globo/isoglobo and ganglio series), steroid biosynthesis, glycerophospholipid metabolism, glycerolipid metabolism. After filtering (R^2^ > 0.6 and p-adjusted < 0.05), 20 lipids and 91 lipid pathway-related transcripts were retained. Pearson correlation between the 91 lipid-pathway related gene transcripts and genome-wide transcripts quantified by RNA-seq (n=16290 transcripts) was computed, and the corresponding p-values were adjusted by Benjamini-Hochberg. After filtering (R^2^ > 0.7; adjusted p value < 0.01), 89 lipid-pathway related gene transcripts and 8299 genome-wide transcripts were retained for partial correlation analysis.

We used full order partial correlations, i.e., correlations between any two variables (e.g., gene, lipids) corrected for all other variables in the dataset, to identify “direct” associations between gene and lipids in response LPS stimulation. The resulting partial correlation network was estimated by Gaussian Graphical Models (GGMs) (64), which provides an improved covariance estimator for a robust inference of large-scale association networks (65); this is implemented in the R package GeneNet (https://strimmerlab.github.io/software/genenet/). GeneNet derived a large partial correlation network connecting all the resolution phase lipids that belong to LC7 and LC8 (39 lipids) and 89 lipid pathway-related transcripts and 8,299 genome-wide transcripts. Using a stringent q-value <10^-12^ threshold to identity significant connections (i.e., significant partial correlations), 34 resolution lipids, 19 lipid pathway-related transcripts and 2,328 other transcripts were retained in a single network, which was then graphically represented using the Cytoscape open-source software platform (66). The genes that had significant partial correlations with globosides and CE were then annotated for pathways using EnrichR (https://maayanlab.cloud/Enrichr/).

## Data Availability Statement

The RNA-seq data was deposited to GEO under GSE112372. MS acquisition files that include MRM transitions, source parameters, column and sample settings used for lipid identifications were deposited to Mendeley Data (https://data.mendeley.com/datasets/ckw7gzrvx6/).

## Ethics Statement

The usage of healthy human blood donor-derived peripheral blood mononuclear cells was reviewed and approved by Imperial College London. The patients/participants provided their written informed consent to participate in this study.

## Author Contributions

Conceptualization: JB, FT, and MW; Methodology: SM, FT, ML, AO, MB, AM-M, J-HK, SJ, BB, HR, EP, and JB; Formal analysis: SM, FT, ML, AO, MB, AM-M, J-HK, SJ, BB, MW; Resources: JB, EP, and MW; Data curation: SJ, ML, EP, AM-M, SM, FT, BB, and JB, Visualization: SJ, ML, EP, SM, AM-M, BB, FT, and JB; Supervision: JB, FT, MW, and EP; Funding acquisition: JB and MW. The manuscript was written by JB, SM, and FT with the help of all co-authors. All authors read and approved the final version of the manuscript.

## Funding

This work was supported by the Medical Research Council UK (MR/M004716/1 and MR/N01121X/1 to JB) and funding support by Duke NUS Singapore (to JB). Work in Singapore Lipidomics Incubator (SM, FT, BB, SJ, ML, MW) was supported by the Life Sciences Institute (LSI) at National University of Singapore, National Research Foundation (NRFI2015-05, NRFSBP-P4) and A*STAR (I1901E0040).

## Conflict of Interest

The authors declare that the research was conducted in the absence of any commercial or financial relationships that could be construed as a potential conflict of interest.

## Publisher’s Note

All claims expressed in this article are solely those of the authors and do not necessarily represent those of their affiliated organizations, or those of the publisher, the editors and the reviewers. Any product that may be evaluated in this article, or claim that may be made by its manufacturer, is not guaranteed or endorsed by the publisher.
