# Supplementary material for: Immunolipidomics Reveals a Globoside Network During the Resolution of Pro-Inflammatory Response in Human Macrophages
Source: Front Immunol. 2022 Jun 30;13:926220. doi: 10.3389/fimmu.2022.926220 (PMC9280915; doi:10.3389/fimmu.2022.926220)
Supplement: Supplementary file 1 [file DataSheet_1.pdf]

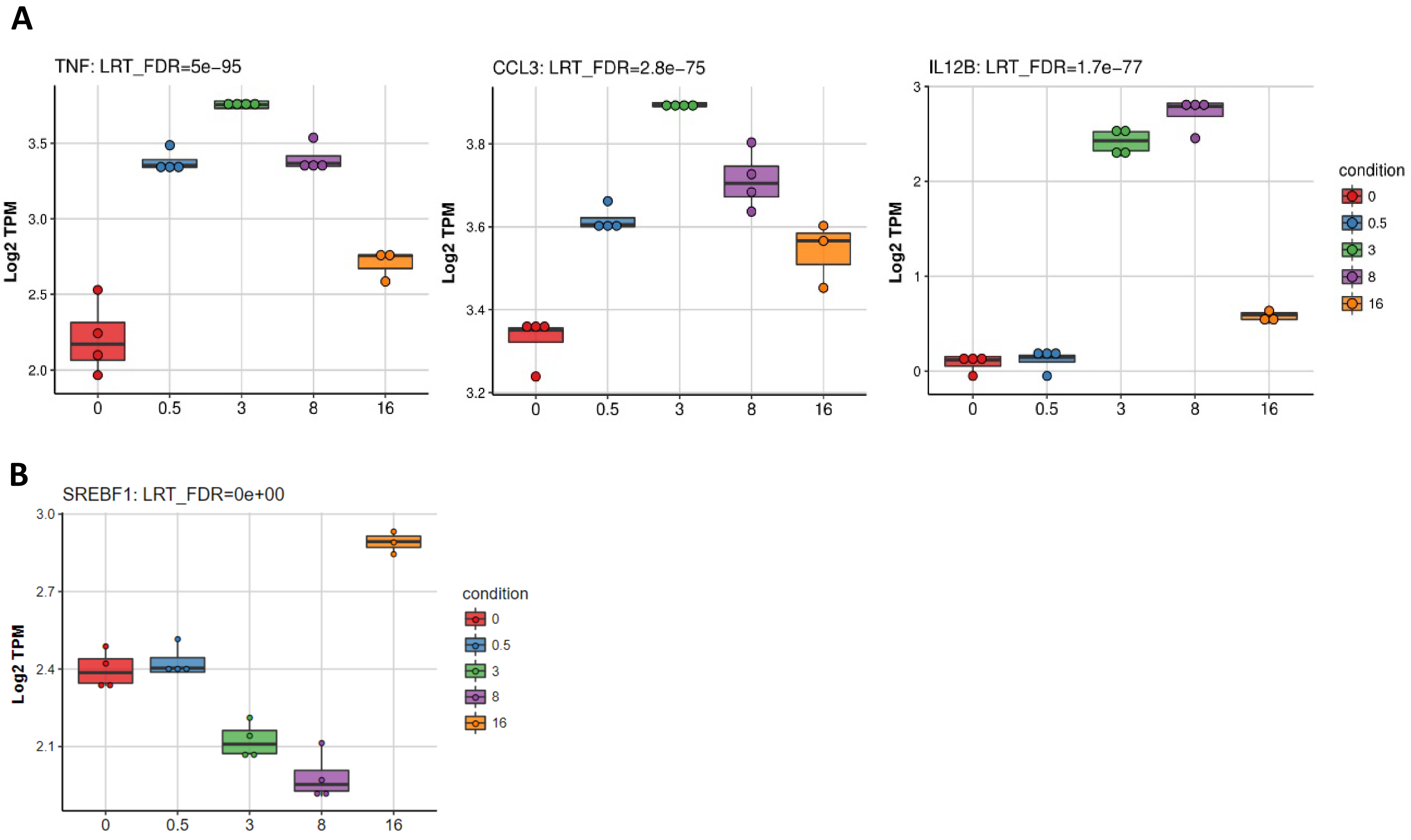

Figure S1. RNA-seq profile (Log2 TPM) of (**A**)*TNF*, *IL12B* and *CCL3* shows a peak at 3h post-LPS stimulation in hMDMs. (**B**) SREBF1 (encoding SREBP1) show a biphasic regulation with distinct pro-inflammatory (3h) and resolution (16h) mRNA expression profiles. At least n=3 biological replicates per time point; TPM, transcript per million; FDR, false discovery rate.

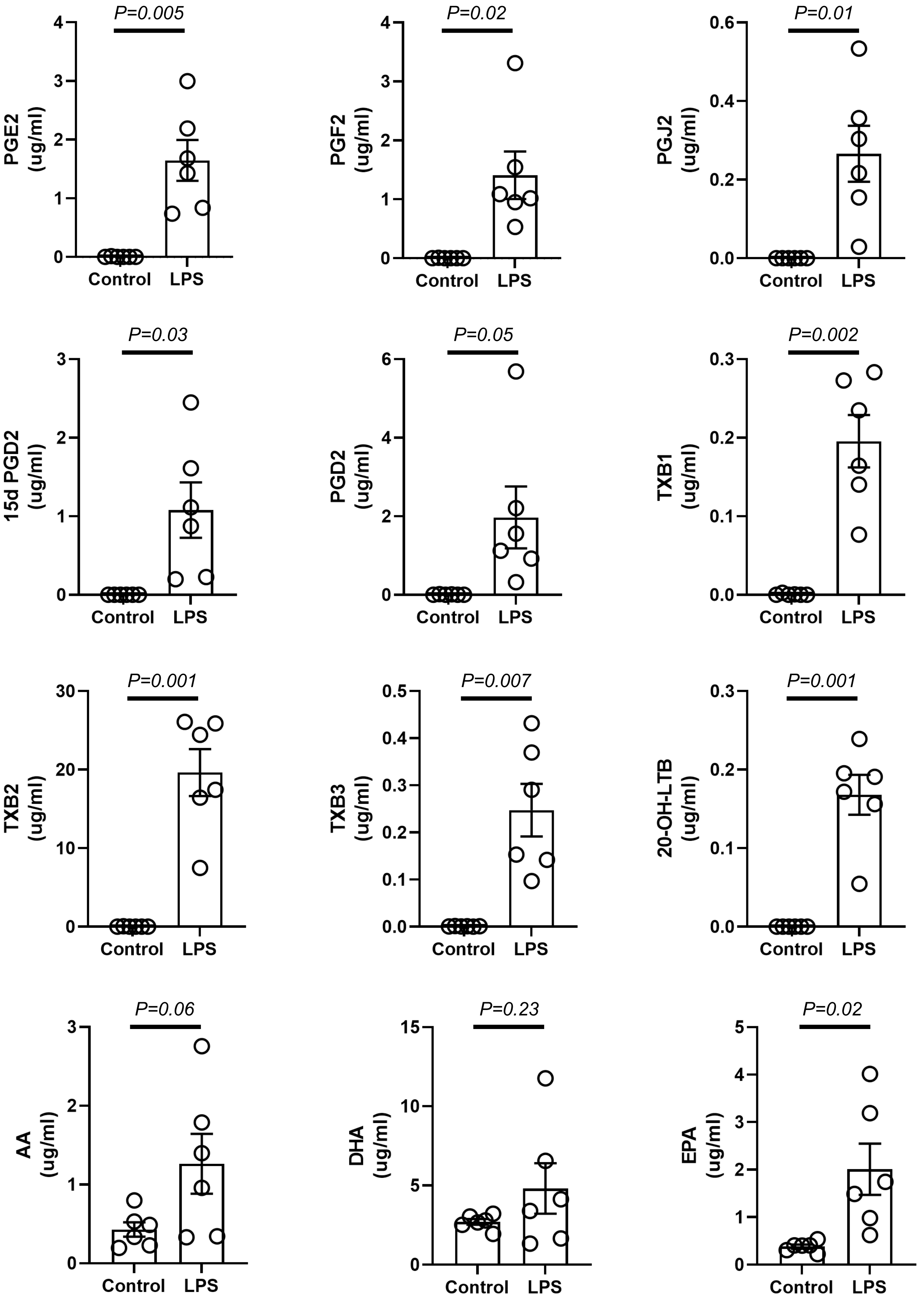

Figure S2. Eicosanoids and AA, DHA and EPA levels measured in the media of control (basal) and 16-stimulated hMDMs. N=6 donors per condition; significance tested by paired t-test.

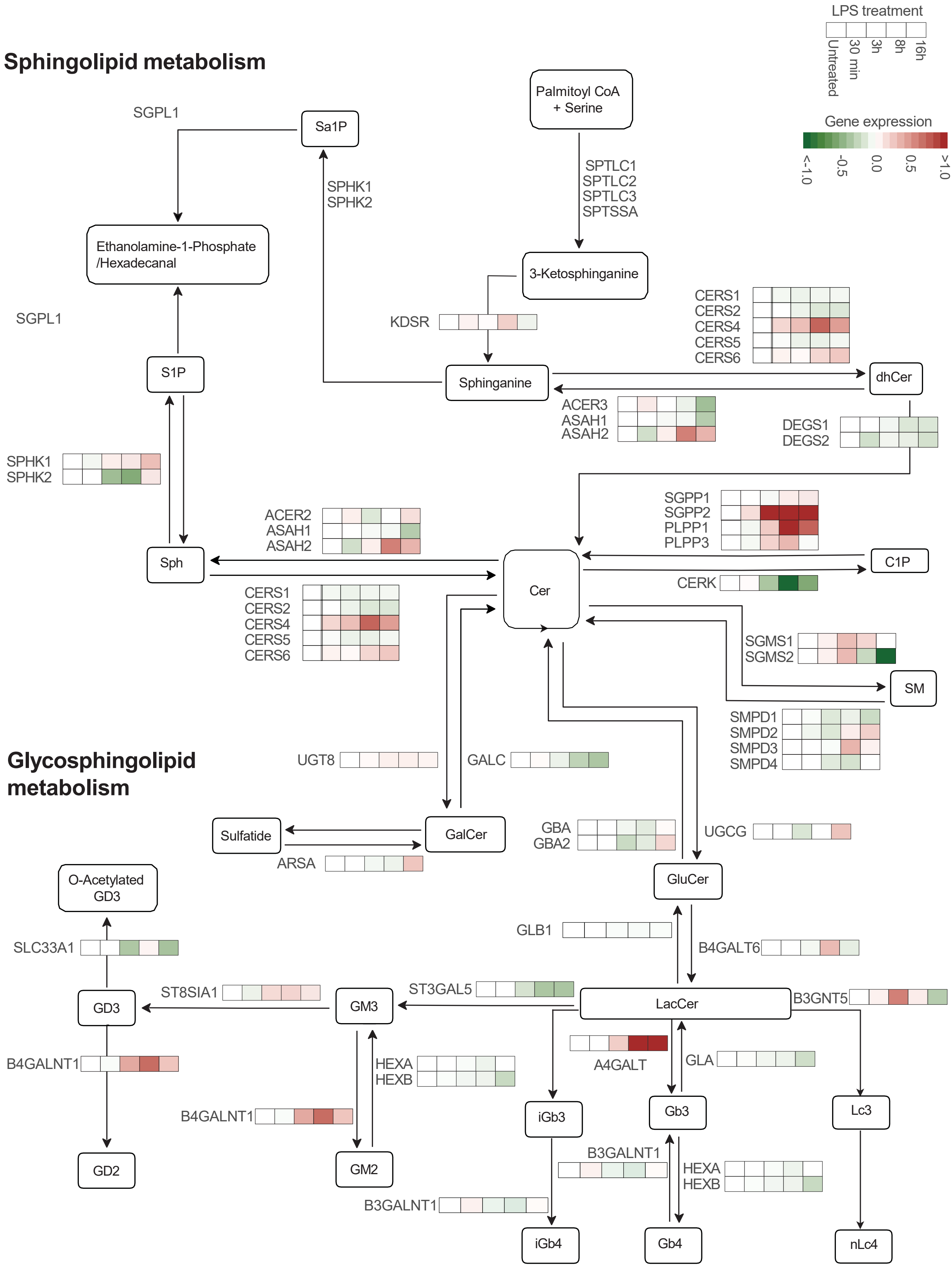

Figure S3. The LPS kinetics of expression of the enzymes in the sphingolipid and glycosphingolipid pathways in human macrophages. All known enzymes of the pathway and their five time-dependent relative expression (untreated, 30 min, 3h, 8h and 16h) are shown.
